# Aflatoxin B1-induced hepatocellular carcinoma in developing countries: Geographical distribution, mechanism of action and prevention

**DOI:** 10.3892/ol.2013.1169

**Published:** 2013-01-31

**Authors:** ABDU SELIM HAMID, ISAIAS GOITOM TESFAMARIAM, YUCHENG ZHANG, ZHEN GUI ZHANG

**Affiliations:** 1 Central Laboratory, China-Japan Union Hospital of Jilin University, Changchun, Jilin 130033;; 2 Institute of Genetics and Cytology, Northeast Normal University, Changchun, Jilin 130033, P.R. China

**Keywords:** aflatoxin B1, hepatocellular carcinoma, DNA adducts, chemopreventive agents, interventions, dietary changes

## Abstract

Hepatocellular carcinoma (HCC) is the most well-known primary liver malignancy worldwide. Its incidence is rising at alarming rates and has become a public concern globally. It is more frequent in developing countries than in industrialized countries with respect to geographical variation, ethnic disparities and socioeconomic status. Dietary exposure to aflatoxins is among the major HCC risk factors. Aflatoxin B1, which is a genotoxic hepatocarcinogen, which presumptively causes cancer by inducing DNA adducts leading to genetic changes in target liver cells. AFB1 is metabolized by cytochrome-P450 enzymes to the reactive intermediate AFB1-8, 9 epoxide (AFBO) which binds to liver cell DNA, resulting in DNA adducts. DNA adducts interact with the guanine bases of liver cell DNA and cause a mutational effect in the *P53* tumor suppressor gene at the codon 249 hotspot in exon 7, which may lead to HCC. Approximately 4.5 billion of the world’s population is exposed to aflatoxin-contaminated food, particularly in low-income countries. Prevention involves treating crops that are susceptible to fungal contamination, appropriate handling of foodstuffs and the use of chemopreventive intervention. Moreover, an integrated network collaboration of different sectors, including public health, agricultural departments and mass media, is required to ensure effective food regulation systems so as to minimize the contamination of food by aflatoxins.

## Contents

1. Introduction
2. Aflatoxin B1
3. Geographical distribution of AFB1
4. Mechanism of action of AFB1 in developing HCC
5. Prevention of HCC associated with AFB1
6. Chemoprevention of AFB1-induced HCC and dietary change
7. Food regulation
8. Conclusion

## 1. Introduction

The incidence of hepatocellular carcinoma (HCC) is rising at an alarming rate and has become a clinical problem worldwide. Data from numerous studies demonstrate that the incidence of HCC has been increasing, with ∼500,000 new cases per year and >600,000 deaths annually (1,2). HCC is the primary liver malignancy, which is the fifth and third leading cause of cancer-related mortality worldwide in females and males, respectively (3,4). HCC is more common in developing countries than in developed countries. However, it has been demonstrated that HCC is also increasing in western countries associated with hepatitis C virus (HCV) infection (5–8). The distribution pattern of HCC reveals geographical variation, ethnic disparities and socioeconomic status with high incidence in Eastern Asia and Africa. There is also an overlap of certain HCC risk factors in their geographical distribution pattern (9–11). Approximately 80% of cases arise in Asia and Africa (12,13). A recent study revealed that China has the highest mortality rate for both males and females, reflecting the prevalence of HBV infection (2), exposure to contaminated food with AFB1 (14) and an increased susceptibility to HCC due to ethnic variation (9).

Among numerous HCC risk factors, hepatitis B virus (HBV) and HCV infections, aflatoxin B1 exposure, heavy alcohol consumption and iron overload are the most documented (15). Epidemiological studies indicate that contamination of food with AFB1 is the major risk factor for human liver cancer (16,17). Areas with a high exposure to AFB1 coincide with areas with a high prevalence of HCC (18,19). AFB1-related HCC is a preventable cancer and thus complete understanding of the mechanism of action of AFB1 and identification of its risk sources are important in minimizing the incidence of AFB1-induced HCC. This may be achieved by establishing interventions, developing chemopreventive agents and adopting dietary changes. In this review, we emphasize the geographical distribution of AFB1-induced HCC, the mechanism of action of AFB1 in HCC development and the prevention of AFB1-induced HCC.

## 2. Aflatoxin B1

**Figure 1:**
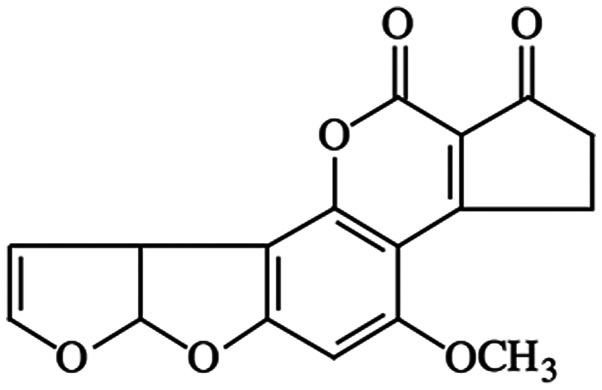
Structure of AFB1 (27).

AFB1 is a mycotoxin produced by the common *Aspergillus flavus* and *Aspergillus parasiticus* which are common and widespread in nature. The mycotoxin is found in foodstuffs, including corn, rice, oil seeds, dried fruits and peanuts, that have been improperly stored in hot, humid and unsanitary conditions (20). It is also found in the milk, meat and eggs of farm animals that feed on aflatoxin-contaminated foods (21,22). Approximately 4.5 billion people are at risk of chronic exposure to aflatoxin-contaminated food. According to the US Food and Drug Administration, AFB1 is considered to be an unavoidable contaminant of food, but nevertheless can be minimized (23). There are four aflatoxins (aflatoxin B1, B2, G1 and G2) that are known to be carcinogenic to both humans and animals, of which aflatoxin B1 (structure shown in Fig. 1) is the most potent hepatotoxic and hepatocarcinogenic agent. AFB1 is well-known to have a range of biological activities, including acute toxicity, teratogenicity, mutagencity and carcinogenicity (24). Reports from epidemiological studies have demonstrated that AFB1 is the most hepatocarcinogenic mycotoxin and the main contributor to the high rate of HCC (25). The International Agency for Research on Cancer (IARC) classified AFB1 as a well-known carcinogenic agent (within group I carcinogens) for HCC. AFB1 is very common in areas such as Southeast Asia and Sub-Saharan Africa. Recently, a survey study conducted in Iran demonstrated that AFB1 was found in a local traditional foodstuff called ‘kashkineh’, which is used as a cold medicine in winter and autumn by inhabitants of certain places in the country (26). Thus, the aforementioned studies demonstrate that AFB1 is found in a variety of foods for both human and animal consumption. Furthermore, its distribution pattern is also correlated with socioeconomic status, and is therefore more common in low-income countries, due to poor sanitation, improper handling of food and ineffective food regulations. Individuals continuously exposed to this toxin through contaminated food grains and animal products may develop both acute hepatotoxicity and HCC.

## 3. Geographical distribution of AFB1

It is well-established that AFB1, a fungal metabolite found in agricultural products, including rice, peanuts, cereals, dried fruits, oil seeds and beers (from barley), is widely found in areas of Southeast Asia and Sub-Saharan Africa. These areas have humid and dry climates that are highly suitable for the proliferation of fungal species, particularly *Aspergillus flavus* and *Aspergillus parasiticus*, which are the main producers of AFB1. Food grains may be contaminated by AFB1 at a number of stages, but is most common when crops are exposed during harvesting and storage, provided that hot and humid weather conditions, improper and unsanitary storage exist for a prolonged period (26,28). A cross-sectional survey conducted by Lewis *et al*(29) aimed to assess the extent of maize contamination in a homegrown market and evaluated the association between the market maize aflatoxin level and an aflatoxicosis outbreak in April 2004, in the most affected regions of Kenya. It was revealed that 55% of the maize was contaminated by AFB1. Moreover, acute aflatoxicosis outbreaks as a consequence of ingesting aflatoxin-contaminated foods have been reported in Kenya, India and Thailand. In West African countries, including Ghana, Togo, Nigeria and Benin, AFB1 is commonly found in both human and animal foods (30–33). These examples suggest that environmental sanitation, correct food storage and effective food regulations are the main challenges in developing countries in preventing and controlling aflatoxicosis, and thereby lowering the incidence of HCC.

## 4. Mechanism of action of AFB1 in developing HCC

**Figure 2:**
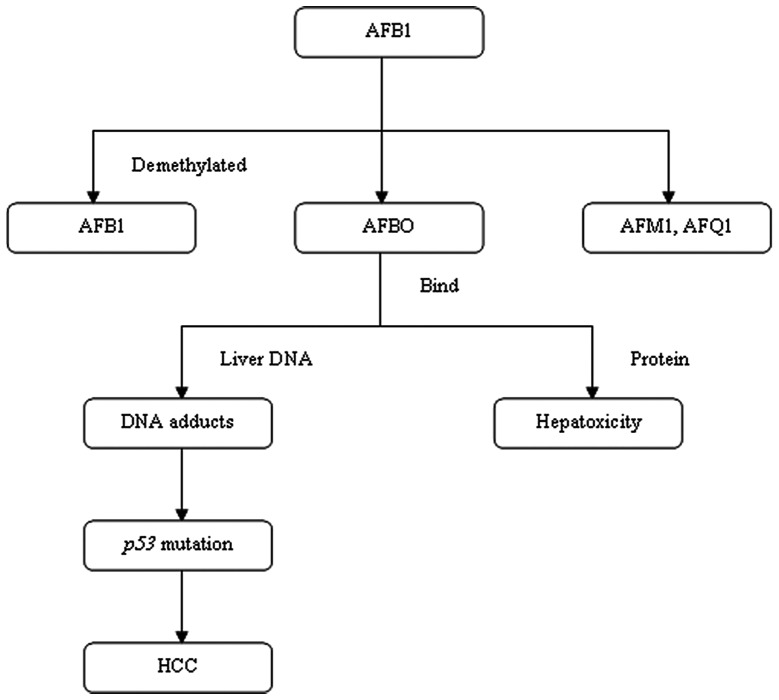
Biotransformation of AFB1, which comprises CYP450-mediated reactions resulting in a highly nucleophilic genotoxic reactive intermediate (AFBO), hydroxylation (to AFM1 and AFQ1) or demethylation (to AFP1). When AFBO binds to liver cell DNA, it causes mutation of *p53* that may lead to HCC. AFBO is also capable of causing aflatoxicosis when it binds to protein amino acids. AFB1, aflatoxin B1; AFBO, AFB1-8, 9 epoxide.

Food mutagens are classified as genotoxic and non-genotoxic agents based on their mechanism of action in inducing cancer (34). Genotoxic agents, which include microcomponents of nutrition, such as aflatoxin, are clearly defined as agents that cause genetic alterations with a mode of action initiating at the DNA level. However, non-genotoxic agents are mostly macrocomponents of nutrition which are presumed to indirectly affect cells through tumor promoters, although they are less defined in their mode of action (34,35). AFB1 is a genotoxic hepatocarcinogen that presumptively causes cancer by inducing DNA adducts, leading to genetic changes in the target cells, which then cause DNA strand breakage, DNA base damage and oxidative damage that may ultimately lead to cancer. DNA adducts arise from chemical modification of the bases in DNA or amino acids in proteins by toxic carcinogenic chemicals (36). Approximately half of human cancers are due to a mutated *Tp53* gene. The mutations affecting *p53* are diverse by their nature and position. For example, mutations such as the transversion in codon 249 [guanine (G) to thymine (T)], which causes an argenine (R) to serine (S) substitution, are present in 50% of HCCs (37,38). This may be due to consumption of AFB1-contaminated food (39,40). Studies in areas with high exposure to AFB1 have demonstrated that AFB1-related HCC is due to *p53* gene mutation; studies from Qidong and Guanxi, China, and South Africa revealed that the mutation occurs at the codon 249 hotspot in exon 7 of the *p53* gene in HCC patients (31,39,41–43). This mutation is termed as 249^ser^, due to the conversion of G to T, which results in R to S mutation in the p53 protein (44). The target organ for metabolism of AFB1 is the liver, where its mechanism of action initiates. Following ingestion with food, AFB1 may be metabolized by cytochrome-P450 enzymes to reactive genotoxic intermediates (aflatoxin B1-8, 9-oxide, AFBO) or hydroxylated (to AFQ1 and AFM1) and demethylated (to AFP1) to become less harmful than AFB1 (as demonstrated in Fig. 2). In order to exert its hepatocarcinogenic effect, AFB1 has to be biotransformed by the cytochrome-P450 enzyme, which results in the production of a reactive intermediate chemical compound, AFBO. This highly reactive genotoxic compound binds to liver cell DNA as a result, and DNA adducts are formed, namely 8, 9-dihydro-8 (N7guanyl)-9-hydroxy-AFB1 (AFB1 N7-Gua) (28,29,35,36). If this is not repaired before DNA replication, the DNA adducts interact with the guanine base of the DNA and cause mutational effects in the *p53* tumor suppressor gene (28,35,45), resulting in hepatocarcinogenesis. Mutated R249Sp53 protein expression may lead to inhibition of apoptosis, inhibition of p53-mediated transcription and stimulation of liver cell growth *in vitro*(37).

**Figure 3:**
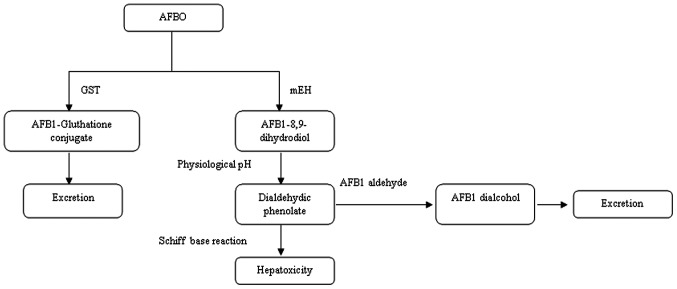
Detoxification pathway of the AFBO intermediate. AFB1, aflatoxin B1; AFBO, AFB1-8, 9 epoxide; GST, glutathionine S-transferase.

Detoxification of the highly genotoxic AFBO intermediate is an essential pathway in order to prevent the formation of DNA adducts. As illustrated in Fig. 3, two potential mechanisms involved in the elimination of AFBO are its conjugation to glutathione by glutathionine S-transferase (GST) and its hydrolysis to dihydrodiol by human microsomal epoxide hydrolase (mEH). Conjugation of AFB1 to glutathione and its subsequent excretion is regarded as an important detoxification pathway in animals (24). In adult mice and rats, the active epoxides are detoxified into their inactive forms, AFB1-glutathione conjugate by GST, particularly by mGSTA3-3 and rGSTA5-5, respectively, and are then degraded (46–48). However, newborn mice, humans and other primates are found to be sensitive to low doses of AFB1. A previous study demonstrated that GST A3 knockout mice became sensitive to both the acute cytotoxic and genotoxic effects of AFB1 (43,48). Alternatively, human microsomal epoxide hydrolase may also be involved in the detoxification of AFBO in the absence of significant GST activity (12,14,37,49–51), by catalyzing it into AFB1-dihydrodiol (52–54). It has also been demonstrated that under physiological conditions, AFB1-dihydrodiol is able to rearrange and form a dialdehyde configuration that is capable of binding to the amine groups in proteins by Schiff base reactions (55).

## 5. Prevention of HCC associated with AFB1

Genetic defects only account for 5–10% of all cancers, whereas environmental factors and lifestyle contribute to 90–95% of cancer cases (56), which implies that cancer may be preventable if we avoid or minimize the risk factors contributing to cancer development (28,35). This requires smoking cessation, increased intake of fruit and vegetables, moderate alcohol consumption, caloric restriction, exercise, avoidance of direct exposure to sunlight, minimal meat consumption, use of whole grains, use of vaccinations and regular check-ups (50,56).

HCC associated with AFB1 is the main concern of public health sectors in developing countries. This is an environmentally preventable risk factor of HCC. However, it remains a bottleneck in countries with poor public health and agricultural systems of safety and food regulation. Effective methods that have ensured minimal contamination of AFB1 in industrialized countries are not able to realistically be implemented in countries with low resources (23). Therefore, practical and effective methods to minimize contamination of human and animal foods by AFB1 ought to be designed for developing countries.

**Figure 4:**
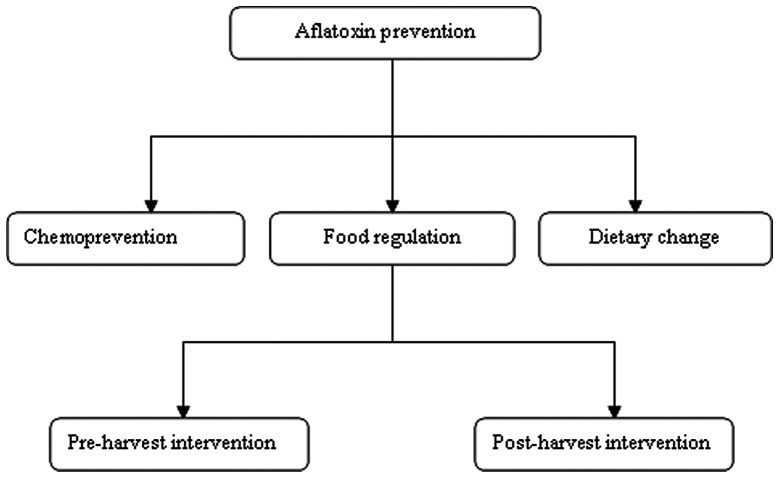
Proposed scheme for aflatoxin prevention.

There are a number of interventions that have been studied to minimize the exposure of aflatoxins at individual or community levels, both termed as primary prevention methods, as well as secondary prevention methods using chemopreventive agents to treat people at high risk of AFB1 exposure (57). Fig. 4 summarizes a proposed scheme that may be more practically applicable and effective in the prevention of AFB1 exposure in low-income countries.

## 6. Chemoprevention of AFB1-induced HCC and dietary change

Cancer chemoprevention is an interventional approach targeting the inhibition or reversal of carcinogenic processes by developing chemopreventive agents (58).

Natural products have potential chemopreventive effects in counteracting AFB1-associated HCC and aflatoxicosis. This is supported by a recent study conducted in mice that evaluated the hepatoprotective effect of the cactus cladode extract (CCE) from cactus *Opuntia ficus indica* on AFB1-induced liver damage. The results demonstrated a chemopreventive effect of CCE against the genotoxicity of AFB1 (59).

Sufficient evidence based on animal model experiments has shown that dietary antioxidants have a therapeutic effect against HCC. It has been well-documented that fruit and vegetables contain anticarcinogenic compounds. A study conducted by Peterson *et al*(60) evaluated the effect of apiaceous vegetables, such as carrots, parsnips, celery and parsley, on human cytochrome-P450 1A2 (hCYP1A2); a liver enzyme known to activate several procarcinogens, including AFB1, was found to have an inhibitory action on hCYP1A2. This suggests that a regular diet including potential protective apiaceous vegetables can decrease the hepatocarcinogenic effect of AFB1. In a study conducted in F344 experimental rats, grapefruits were demonstrated to have a protective potential against AFB1-induced liver DNA damage through a reduction in hepatic CYP3A activity (61).

A coffee-dependent induction of enzymes involved in xenobiotic detoxification processes was observed in rat liver and primary hepatocytes, and it was concluded that an induction of detoxifying enzymes, GSTs and aldo-keto reductase (AKR) by coffee may also provide protection against both genotoxicity and cytotoxicity of AFB1, which are likely to act synergistically in the process of liver cancer development (62).

In a recent study using human hepatocytes in primary culture it was demonstrated that the naturally occurring biologically active compounds phenethyl isothiocyanate (PEITC) and sulforaphane (SFN) have chemoprotective effects against AFB-DNA adduct formation (63).

## 7. Food regulation

Dietary exposure to carcinogens and mutagens depends on individual consumption patterns, which change with age, availability of food and lifestyle (64). Human and animal foods are commonly contaminated by aflatoxins during plantation, crop maturation, harvesting and storage. To prevent hepatotoxicity and hepatocellular carcinogenesis caused by AFB1, establishment of long-term interventions such as comprehensive food safety programs need to be implemented. Interventions ought to be at an individual or community level. These interventions have to target both market vendors and local farmers in order to prevent or minimize long-term exposure to aflatoxins, and thereby decrease HCC incidence rates (29,65). Pre-harvest intervention includes the introduction of crops resistant to fungal infection or aflatoxin biosynthesis, and the spraying of insecticides and fungicides (57,58). A community-based survey, which was conducted in Guinea by Turner *et al*, aimed to reduce exposure to AFB1 by post-harvest intervention; the use of low-technology approaches was suggested. These approaches included hand sorting, drying on mats instead of on the ground, complete sun drying, spraying insecticide on the store floor at the start of storage, along with regular intervals afterwards, and refraining from the use of plastic or synthetic materials for storage that promote humidity (66).

## 8. Conclusion

HCC is the most well-known primary liver malignancy and is a major health problem worldwide. From an epidemiological point of view, AFB1 is the most hepatocarcinogenic agent and a main contributor to the high rate of HCC. AFB1 is considered to be an unavoidable contaminant of food, but nevertheless can be minimized. Dietary exposure to AFB1 is very common in developing countries. AFB1 is found in a variety of foodstuffs for both human and animal consumption. AFB1-associated HCC is preventable if we avoid exposure to aflatoxins by implementing numerous measures, including dietary changes, chemoprevention and optimal food safety and regulation. It has also been well documented that fruit and vegetables have anticarcinogenic compounds. Therefore, practical and effective methods to minimize contamination of human and animal foods by AFB1 ought to be designed for developing countries. Integrated network collaborations of different sectors, including public health, agricultural departments and mass media, are required to ensure effective food regulation systems so as to minimize contamination of food by aflatoxins. By training farmers how to implement locally applicable interventions and increasing awareness of dietary changes and sanitation, prevention methods may be initiated at a local level.

## References

[1] Llovet JM, Burroughs A, Bruix J (2003). Hepatocellular carcinoma. Lancet.

[2] Lord R, Suddle A, Ross PJ (2011). Emerging strategies in the treatment of advanced hepatocellular carcinoma: the role of targeted therapies. Int J Clin Pract.

[3] El-Serag HB, Rudolph KL (2007). Hepatocellular carcinoma: epidemiology and molecular carcinogenesis. Gastroenterology.

[4] Ferlay J, Shin HR, Bray F, Forman D, Mathers C, Parkin DM (2010). Estimates of worldwide burden of cancer in 2008: GLOBOCAN 2008. Int J Cancer.

[5] El-Serag HB, Mason AC (1999). Rising incidence of hepatocellular carcinoma in the United States. N Engl J Med.

[6] Altekruse SF, McGlynn KA, Reichman ME (2009). Hepatocellular carcinoma incidence, mortality, and survival trends in the United States from 1975 to 2005. J Clin Oncol.

[7] Nguyen MH, Whittemore AS, Garcia RT (2004). Role of ethnicity in risk for hepatocellular carcinoma in patients with chronic hepatitis C and cirrhosis. Clin Gastroenterol Hepatol.

[8] Farazi PA, DePinho RA (2006). Hepatocellular carcinoma pathogenesis: from genes to environment. Nat Rev Cancer.

[9] Ward E, Jemal A, Cokkinides V (2004). Cancer disparities by race/ethnicity and socioeconomic status. CA Cancer J Clin.

[10] McCracken M, Olsen M, Chen MS (2007). Cancer incidence, mortality, and associated risk factors among Asian Americans of Chinese, Filipino, Vietnamese, Korean, and Japanese ethnicities. CA Cancer J Clin.

[11] Röcken C, Carl-McGrath S (2001). Pathology and pathogenesis of hepatocellular carcinoma. Dig Dis.

[12] McGlynn KA, London WT (2005). Epidemiology and natural history of hepatocellular carcinoma. Best Pract Res Clin Gastroenterol.

[13] Sherman M (2005). Hepatocellular carcinoma: epidemiology, risk factors, and screening. Semin Liver Dis.

[14] London WT, Evans AA, McGlynn K (1995). Viral, host and environmental risk factors for hepatocellular carcinoma: a prospective study in Haimen City, China. Intervirology.

[15] Chen CJ, Yu MW, Liaw YF (1997). Epidemiological characteristics and risk factors of hepatocellular carcinoma. J Gastroenterol Hepatol.

[16] Madden CR, Finegold MJ, Slagle BL (2002). Altered DNA mutation spectrum in aflatoxin b1-treated transgenic mice that express the hepatitis B virus x protein. J Virol.

[17] Li Y, Su JJ, Qin LL, Yang C, Ban KC, Yan RQ (1999). Synergistic effect of hepatitis B virus and aflatoxin B1 in hepatocarcinogenesis in tree shrews. Ann Acad Med Singapore.

[18] Montalto G, Cervello M, Giannitrapani L, Dantona F, Terranova A, Castagnetta LA (2002). Epidemiology, risk factors, and natural history of hepatocellular carcinoma. Ann NY Acad Sci.

[19] Groopman JD, Scholl P, Wang JS (1996). Epidemiology of human aflatoxin exposures and their relationship to liver cancer. Prog Clin Biol Res.

[20] Baydar T, Engin AB, Girgin G, Aydin S, Sahin G (2005). Aflatoxin and ochratoxin in various types of commonly consumed retail ground samples in Ankara, Turkey. Ann Agric Environ Med.

[21] Bennett JW, Klich M (2003). Mycotoxins. Clin Microbiol Rev.

[22] Fink-Gremmels J (1999). Mycotoxins: their implications for human and animal health. Vet Q.

[23] Williams JH, Phillips TD, Jolly PE, Stiles JK, Jolly CM, Aggarwal D (2004). Human aflatoxicosis in developing countries: a review of toxicology, exposure, potential health consequences, and interventions. Am J Clin Nutr.

[24] McLean M, Dutton MF (1995). Cellular interactions and metabolism of aflatoxin: an update. Pharmacol Ther.

[25] Wang JS, Huang T, Su J (2001). Hepatocellular carcinoma and aflatoxin exposure in Zhuqing Village, Fusui County, People’s Republic of China. Cancer Epidemiol Biomarkers Prev.

[26] Mardani M, Rezapour S, Rezapour P (2011). Survey of aflatoxins in Kashkineh: A traditional Iranian food. Iran J Microbiol.

[27] Banerjee S, Brown KL, Egli M, Stone MP (2011). Bypass of aflatoxin B1 adducts by the Sulfolobus solfataricus DNA polymerase IV. J Am Chem Soc.

[28] Obuseh FA, Jolly PE, Kulczycki A (2011). Aflatoxin levels, plasma vitamins A and E concentrations, and their association with HIV and hepatitis B virus infections in Ghanaians: a cross-sectional study. J Int AIDS Soc.

[29] Lewis L, Onsongo M, Njapau H (2005). Aflatoxin contamination of commercial maize products during an outbreak of acute aflatoxicosis in eastern and central Kenya. Environ Health Perspect.

[30] Oyelami OA, Maxwell SM, Adeoba E (1996). Aflatoxins and ochratoxin A in the weaning food of Nigerian children. Ann Trop Paediatr.

[31] Egal S, Hounsa A, Gong YY (2005). Dietary exposure to aflatoxin from maize and groundnut in young children from Benin and Togo, West Africa. Int J Food Microbiol.

[32] Kpodo K, Thrane U, Hald B (2000). Fusaria and fumonisins in maize from Ghana and their co-occurrence with aflatoxins. Int J Food Microbiol.

[33] Awuah RT, Kpodo KA (1996). High incidence of *Aspergillus flavus* and aflatoxins in stored groundnut in Ghana and the use of a microbial assay to assess the inhibitory effects of plant extracts on aflatoxin synthesis. Mycopathologia.

[34] Sugimura T (2000). Nutrition and dietary carcinogens. Carcinogenesis.

[35] Sutandyo N (2010). Nutritional carcinogenesis. Acta Med Indones.

[36] Sharma RA, Farmer PB (2004). Biological relevance of adduct detection to the chemoprevention of cancer. Clin Cancer Res.

[37] Martin J, Dufour JF (2008). Tumor suppressor and hepatocellular carcinoma. World J Gastroenterol.

[38] Jackson PE, Kuang SY, Wang JB (2003). Prospective detection of codon 249 mutations in plasma of hepatocellular carcinoma patients. Carcinogenesis.

[39] Bressac B, Kew M, Wands J, Ozturk M (1991). Selective G to T mutations of p53 gene in hepatocellular carcinoma from southern Africa. Nature.

[40] Montesano R, Hainaut P, Wild CP (1997). Hepatocellular carcinoma: from gene to public health. J Natl Cancer Inst.

[41] Hsu IC, Metcalf RA, Sun T, Welsh JA, Wang NJ, Harris CC (1991). Mutational hotspot in the p53 gene in human hepatocellular carcinomas. Nature.

[42] Ozturk M (1991). p53 mutation in hepatocellular carcinoma after aflatoxin exposure. Lancet.

[43] Stern MC, Umbach DM, Yu MC, London SJ, Zhang ZQ, Taylor JA (2001). Hepatitis B, aflatoxin B(1), and p53 codon 249 mutation in hepatocellular carcinomas from Guangxi, People’s Republic of China, and a meta-analysis of existing studies. Cancer Epidemiol Biomarkers Prev.

[44] Nogueira JA, Ono-Nita SK, Nita ME (2009). 249 TP53 mutation has high prevalence and is correlated with larger and poorly differentiated HCC in Brazilian patients. BMC Cancer.

[45] Wang JS, Groopman JD (1999). DNA damage by mycotoxins. Mutat Res.

[46] Eaton DL, Gallagher EP (1994). Mechanisms of aflatoxin carcinogenesis. Annu Rev Pharmacol Toxicol.

[47] Gallagher EP, Kunze KL, Stapleton PL, Eaton DL (1996). The kinetics of aflatoxin B1 oxidation by human cDNA-expressed and human liver microsomal cytochromes P450 1A2 and 3A4. Toxicol Appl Pharmacol.

[48] Ilic Z, Crawford D, Vakharia D, Egner PA, Sell S (2010). Glutathione-S-transferase A3 knockout mice are sensitive to acute cytotoxic and genotoxic effects of aflatoxin B1. Toxicol Appl Pharmacol.

[49] Dash B, Afriyie-Gyawu E, Huebner HJ (2007). Determinants of the variability of aflatoxin-albumin adduct levels in Ghanaians. J Toxicol Environ Health A.

[50] Eaton DL, Bammler TK, Kelly EJ (2001). Interindividual differences in response to chemoprotection against aflatoxin-induced hepatocarcinogenesis: implications for human biotransformation enzyme polymorphisms. Adv Exp Med Biol.

[51] Kirk GD, Turner PC, Gong Y (2005). Hepatocellular carcinoma and polymorphisms in carcinogen-metabolizing and DNA repair enzymes in a population with aflatoxin exposure and hepatitis B virus endemicity. Cancer Epidemiol Biomarkers Prev.

[52] Smela ME, Currier SS, Bailey EA, Essigmann JM (2001). The chemistry and biology of aflatoxin B(1): from mutational spectrometry to carcinogenesis. Carcinogenesis.

[53] Wild CP, Turner PC (2002). The toxicology of aflatoxins as a basis for public health decisions. Mutagenesis.

[54] Guengerich FP, Johnson WW, Shimada T, Ueng YF, Yamazaki H, Langouët S (1998). Activation and detoxication of aflatoxin B1. Mutat Res.

[55] Sabbioni G, Skipper PL, Büchi G, Tannenbaum SR (1987). Isolation and characterization of the major serum albumin adduct formed by aflatoxin B1 in vivo in rats. Carcinogenesis.

[56] Anand P, Kunnumakkara AB, Sundaram C (2008). Cancer is a preventable disease that requires major lifestyle changes. Pharm Res.

[57] Wild CP, Hall AJ (2000). Primary prevention of hepatocellular carcinoma in developing countries. Mutat Res.

[58] Gescher AJ, Sharma RA, Steward WP (2001). Cancer chemoprevention by dietary constituents: a tale of failure and promise. Lancet Oncol.

[59] Brahmi D, Bouaziz C, Ayed Y, Ben Mansour H, Zourgui L, Bacha H (2011). Chemopreventive effect of cactus *Opuntia ficus indica* on oxidative stress and genotoxicity of aflatoxin B1. Nutr Metab (Lond).

[60] Peterson S, Lampe JW, Bammler TK, Gross-Steinmeyer K, Eaton DL (2006). Apiaceous vegetable constituents inhibit human cytochrome P-450 1A2 (hCYP1A2) activity and hCYP1A2-mediated mutagenicity of aflatoxin B1. Food Chem Toxicol.

[61] Miyata M, Takano H, Guo LQ, Nagata K, Yamazoe Y (2004). Grapefruit juice intake does not enhance but rather protects against aflatoxin B1-induced liver DNA damage through a reduction in hepatic CYP3A activity. Carcinogenesis.

[62] Cavin C, Marin-Kuan M, Langouët S (2008). Induction of Nrf2-mediated cellular defenses and alteration of phase I activities as mechanisms of chemoprotective effects of coffee in the liver. Food Chem Toxicol.

[63] Gross-Steinmeyer K, Stapleton PL, Tracy JH, Bammler TK, Strom SC, Eaton DL (2010). Sulforaphane- and phenethyl isothiocyanate-induced inhibition of aflatoxin B1-mediated genotoxicity in human hepatocytes: role of GSTM1 genotype and CYP3A4 gene expression. Toxicol Sci.

[64] Sotomayor RE, Washington M, Nguyen L, Nyang’anyi R, Hinton DM, Chou M (2003). Effects of intermittent exposure to aflatoxin B1 on DNA and RNA adduct formation in rat liver: dose-response and temporal patterns. Toxicol Sci.

[65] McGlynn KA, Hunter K, LeVoyer T (2003). Susceptibility to aflatoxin B1-related primary hepatocellular carcinoma in mice and humans. Cancer Res.

[66] Turner PC, Sylla A, Gong YY (2005). Reduction in exposure to carcinogenic aflatoxins by postharvest intervention measures in west Africa: a community-based intervention study. Lancet.

